# Plug the pit: a surgical technique for optic disc pit

**DOI:** 10.22336/rjo.2021.74

**Published:** 2021

**Authors:** Davide Borroni, Chiara Bonzano, Rahul Rachwani Anil, María García Lorente, Carlos Rocha de Lossada, Francisco Zamorano Martín, Hussain Ahmad Khaqan

**Affiliations:** * The Veneto Eye Bank Foundation, Venice, Italy; ** Clinica Oculistica, Department of Neuroscience, Rehabilitation, Ophthalmology, Genetics, Maternal and Child Health (DiNOGMI), University of Genoa; *** Hospital Regional de Málaga, Departamento de Oftalmología, Málaga, Spain; **** Hospital Costa del Sol, Departamento de Oftalmología, Marbella, Spain; ***** University Hospital Virgen de las Nieves, Granada, Spain; ****** Lahore General Hospital, Post Graduate Medical Institute, Lahore, Pakistan

**Keywords:** maculopathy, optic disc pit, punctal plug

## Abstract

**Purpose:** To present a recently described surgical technique for the treatment of optic disc pit (ODP) and evaluate its outcomes.

**Methods:** A patient presented with refractory serous macular detachment and secondary full thickness macular hole associated with ODP, for which he had already undergone pars-plana vitrectomy with internal limiting membrane peeling and autologous serum application over the optic disc pit. A recently described surgical technique was carried out to treat this case. In this procedure, a silicone punctal plug was used to close the ODP. The macular hole was closed with a human amniotic membrane graft. Endotamponade was carried out with 1000cs silicone oil.

**Results:** Postoperatively, the serous macular detachment subsided and the punctal plug and amniotic membrane graft were *in situ*. Patient’s visual acuity improved from counting fingers to 6/38 at one year postoperative.

**Conclusion:** This technique appears to be safe and effective in resolving long standing serous macular detachment associated with ODP, which was refractory to the conventional intervention. However, more cases and longer follow-ups are needed to affirm the safety and efficacy of this recently described procedure.

## Introduction

Optic disc pit (ODP) is a congenital disc anomaly of the optic nerve head, with an incidence of approximately 1:10,000 people [**1**,**2**]. It can occur in isolation or in association with the optic disc coloboma [**3**]. ODP is unilateral in most of the cases, and bilateral incidence has been observed in 15% of the cases [**1**,**2**]. There is no gender predilection [**1**]. Treatment of ODP associated maculopathy is still controversial.

Spontaneous resolution occurs in 25% of the cases. Many surgeons have described different techniques, including injection of intravitreal gases [**4**], laser photocoagulation along the temporal aspect of the optic disc [**5**], pars-plana vitrectomy (PPV) with internal limiting membrane (ILM) peeling and gas tamponade [**6**], insertion of ILM into the ODP [**7**], and the use of autologous serum to fill the ODP [**8**]. All these strategies have produced inconsistent results.

We present a patient with ODP and secondary macular hole, which was assessed with a recently described surgical technique [**9**]. We chose to close the ODP with a silicone punctal plug. An amniotic membrane graft was used to close the macular hole.

## Methods

**Fig. 1:**
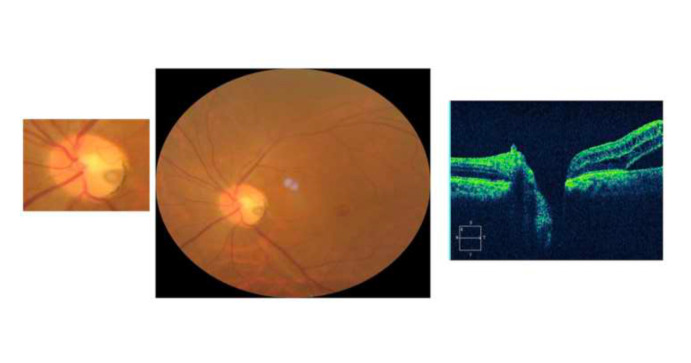
Fundus picture and OCT images before surgery showing persistent optic disc pit with associated maculopathy and secondary macular hole

A 45-year-old Asian patient presented to our outpatient department due to a relapsed ODP and concomitant macular hole (**Fig. 1**). The size of the ODP was 0.4 mm and the macular hole width was 621 µm. Past ocular history of the patient included a 23G PPV, ILM peeling and autologous serum filling for ODP and associated serous macular detachment and macular hole six months prior to the visit to our department.

**Fig. 2:**
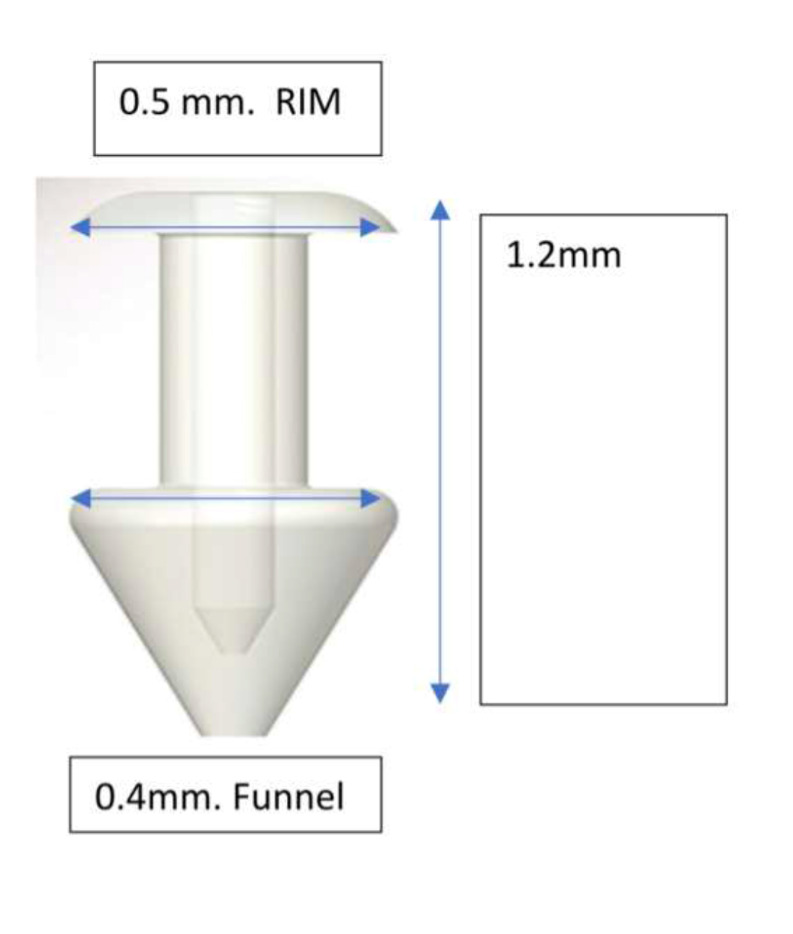
Medium sized silicone punctal plug that was used in this procedure and its dimensions (Veraplug, Lacrivera)

We planned to close the ODP with a silicone punctal plug and the macular hole with human amniotic membrane graft, as well as phacoemulsification surgery as the patient also presented cataract. Visual acuity (VA) in the right eye (RE) was counting fingers (CF), and 20/20 in the left eye (LE). Fundus photographs were taken using Topcon fundus camera to document the findings. Optical Coherence Tomography (OCT) was carried out using Cirrus 500 OCT (Carl Zeiss Meditec) to measure the size of macular hole and the ODP. Specific measurements included ODP diameter and width in order to choose an appropriate plug size. A medium sized VERA® punctum plug was used, and dimensions of the plug size were 1.2 mm in length, 0.5 mm rim diameter and 0.4 mm funnel diameter. Plug diameter (0.5 mm) was 0.1 mm wider than the widest pit diameter (0.4 mm) (**Fig. 2**). 

The intervention was approved by the ethical review board committee. Informed consent was obtained from the patient and the study followed the declaration of Helsinki. 

*The key steps of the surgical procedure*

Three Standard pars-plana ports were inserted using 23-G valved trocar system (Dutch Ophthalmic Research Laboratory, DORC). Fluid to air exchange was performed with the air pressure set at 50 mmHg, using 23-gauge flute needle held nasal to the disc. With the help of a 23-G end-grasping forceps, silicone punctal plug (Vera Plug Small, Lacrivera) (**Fig. 2**) was grasped. Once in the vitreous cavity, the punctal plug was inserted into the optic disc pit. There were no complications in inserting the plug through the 23G port.

We used human amniotic membrane graft to close the full thickness secondary to the macular hole. The prepared amniotic membrane graft was laid flat on a sterilized paper and cut to an appropriate size using Vannas scissors. The amniotic membrane graft was held with 23-G end grasping forceps, placed over and slightly dipped into the macular hole. Endotamponade consisting of 1000cs silicone oil was carried out to ensure amniotic membrane and punctal plug positioning as dislodging may happen with gas tamponade. Vitrectomy ports were sutured with Vicryl 6/0.

## Results

**Fig. 3:**
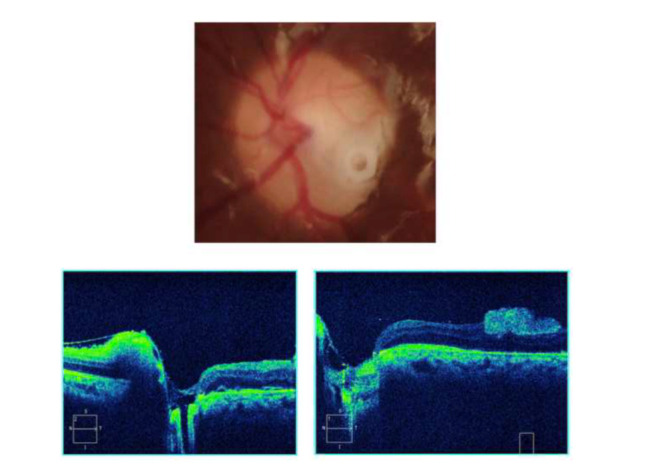
Postoperative fundus picture and OCT images showing punctal plug well placed in the disc pit, and amniotic membrane graft filling the macular hole. Serous macular detachment has subsided

On the first postoperative day, punctal plug was well in place in the ODP, and the amniotic membrane graft covered the macular hole entirely (**Fig. 3**). No preoperative or postoperative complications were observed.

Silicon oil extraction was performed one month postoperative and VA in the RE was 20/100 at one year follow up. The amniotic membrane graft successfully closed the macular hole and the punctal plug sealed the optic disc pit.

## Discussion

Pars plana vitrectomy (PPV) is currently being used to address ODP maculopathy. The induction of posterior vitreous detachment and removal of the vitreous eliminates one hypothesized source of subretinal fluid, as well as relieves the traction on the macula [**6**].

We used this innovative technique as ODP has no standard treatment. We chose this method as this was a relapsed case, and in the first procedure, amniotic membrane was used to close the pit with unsuccessful results. Other options, such as autologous serum and scleral tissue graft have been described to treat ODP [**10**-**12**].

The reason why punctum plug is a viable option is because it plugs the pit thanks to its funnel shaped tip rather than closes the pit. It is easy to insert, biocompatible, inexpensive, and moreover, it is already designed, hence readily available. On the other hand, amniotic membrane and scleral tissue need a graft preparation step and therefore are more tedious techniques. To our understanding, punctal plugs may not harm the nerve fibers by compression as the pit walls are mostly covered with glial tissue [**12**]. 

The punctal plug may dislodge. In this case, it may be difficult to find and it could traumatize the retina or crystalline lens in phakic patients. Nevertheless, this is most likely to occur if a gas is used as a tamponade agent. Moreover, amniotic membrane and scleral grafts may also produce these complications [**12**,**13**]. 

Deltour et al. [**9**] recently published a case report of a patient undergoing punctal plug positioning to plug an ODP after two prior unsuccessful surgeries, specifically, two surgeries consisting of 25G PPV, ILM peeling and C2F6 gas tamponade. As opposed to our case, in the case they presented, the patient did not present macular hole. Also, the scleral port was enlarged to 20G for plug insertion into the vitreous cavity. Moreover, the patient did not improve in VA, it rather remained stable at 20/800.

We believe that although this technique has already been described, there is only one single case reported in literature, and, as opposed to our case, the patient did not show improvement in VA even though he did not even not associate a macular hole like in our case. Silicone punctal plug provides a physical and stronger barrier between the subarachnoid and subretinal space. The main limitation of our study was that we only presented a single case. Nevertheless, given the rarity of the condition and the challenge that relapsed ODP is to the retinal surgeon, we believe that this case adds a piece of scientific knowledge to the outcomes of this procedure. Further research, if possible, a clinical trial, could be interesting to compare this technique with the other alternatives.

## Conclusion

The proposed technique appeared to be safe and effective in closing the optic disc pit.

**Conflicts of Interest statement**

The authors declare that there are no conflicts of interest.

**Informed Consent and Human and Animal Rights statement**

Informed consent has been obtained from all individuals included in this study.

**Authorization for the use of human subjects**

Ethical approval: The research related to human use complies with all the relevant national regulations, institutional policies, is in accordance with the tenets of the Helsinki Declaration, and has been approved by the review board of The Veneto Eye Bank Foundation, Venice, Italy.

**Acknowledgments**

None.

**Sources of Funding**

None.

**Financial Disclosure(s)**

None.
